# Relation between learning process and morphology of transport tube network in plasmodium of *Physarum polycephalum*

**DOI:** 10.3389/fcell.2023.1249165

**Published:** 2023-11-10

**Authors:** Emiri Yoneoka, Atsuko Takamatsu

**Affiliations:** Department of Electrical Engineering and Bioscience, Waseda University, Tokyo, Japan

**Keywords:** slime mold, memory, habituation, transport tube, network, *Physarum polycephalum*

## Abstract

The question of whether a single-celled organism without a brain could have functions such as learning and memory has been the subject of much debate in recent years. The plasmodium of the true slime mold, *Physarum polycephalum*, is an ideal model organism for such a question. The plasmodium exhibits behaviors that resemble intelligence, including solving mazes, mimicking optimal rail transportation networks, predicting the weather, and solving traveling salesman problems. In addition, the plasmodium has recently been shown to have the simplest form of learning: habituation. In the experiments in which plasmodia were repeatedly allowed to cross bridges containing aversive chemicals, the habituation behavior has been confirmed. It has been shown that the habituation process involves chemicals that are stored internally. However, it is not clear how these chemicals result in change in the behavior of plasmodium during habituation learning. This study focused on the transport tube network formed in plasmodium during the above experiments. Then, the role of the network morphology in the habituation learning process was investigated. The results showed that the network morphology changes from tree to mesh type during habituation learning, and disrupting the learned network reduces habituation behavior. In addition, it was shown that the thickness oscillation frequency depends on the network morphology. The study found that in the plasmodium of *P. polycephalum*, a primitive organism without a brain, transport tube networks, instead of neuronal networks, play an important role in habituation learning and the resulting decision making.

## 1 Introduction

The question of whether single-celled organisms without brains have functions such as learning and memory has been much debated in recent years, and the importance of this question has been widely recognized (Dussutour, 2021; Gunawardena, 2022; Wright et al., 2023). Although the definition of learning has long been discussed for animals with brains and nervous systems, it can be extended to single-celled organisms as follows: “learning” is a change in the internal state of a system in response to external stimuli, followed by an appropriate change in the behavior in response to the same stimuli (Barron et al., 2015). From this perspective, the learning function has been reported in a variety of single-celled organisms; examples include *E. coli* (Yi et al., 2000), *Stentor* (Rajan et al., 2023), *Spirostomum* (Eisenstein et al., 1982), and *Paramecium* (French, 1940; Hanzel and Rucker, 1972).

In this context, the plasmodium of the true slime mold, *Physarum polycephalum*, is an ideal model organism. Although it is a single-celled organism, it behaves as if it has “intelligence” in a diorama environment artificially constructed in a laboratory (Nakagaki, 2021). After Nakagaki et al. demonstrated in 2000 that the plasmodium of *P. polycephalum* can solve mazes (Nakagaki et al., 2000a), a variety of important studies relating to their “intelligence” have been conducted. The slime mold can find the shortest path (Nakagaki et al., 2004), optimize railway networks (Tero et al., 2010; Watanabe et al., 2011), predict weather (Saigusa et al., 2008), solve traveling salesman problems (Zhu et al., 2013; Zhu et al., 2018), and more.

The plasmodium is a vegetative form of the true slime mold of *P. polycephalum*. It is a very large amoeboid cell, ranging in size from a few millimeters to several meters. It crawls around in the environment while changing its shape (William et al., 1986). Because the plasmodium has many nuclei within its single-cell body, it can survive even if part of it is separated from the rest of the cell. Then, the separate parts behave as individuals. Meanwhile, there is a network structure of transport tubes, i.e., veins, inside the cell body (Takamatsu et al., 2017). These tubes are responsible for transporting the contents of the cell, cytoplasm (including the nucleus, nutrients, and oxygen), throughout the body. This is an essential structure for the plasmodium that must maintain a large cell body. The outer part of the transport tube is a harder structure consisting of gel, while the inner part consists of a softer material, cytosol. As the plasmodium crawls and spreads through the environment, the hard and soft parts can change their states with each other through sol-gel transformation (Kamiya, 1959). This allows the transport tube network to change shape easily, resulting in an efficient network that responds to the surrounding environment (Takamatsu et al., 2009; Tero et al., 2010; Ito et al., 2011; Takamatsu et al., 2017). For example, in an environment with aversive chemicals or under starvation, the network forms a dendritic or tree structure; in a nutrient-rich environment, it forms a mesh structure with a thin sheet-like structure at the front (Takamatsu et al., 2009).

Recently, studies have directly examined the learning function in plasmodium of *P. polycephalum* (Boisseau et al., 2016; Vogel and Dussutour, 2016; Boussard et al., 2019). These studies have focused on the simplest form of learning, habituation. Habituation, classified as non-associative learning, is a phenomenon in which the response to repeated stimuli decreases. To determine this phenomenon as a form of learning, nine criteria were initially established (Thompson and Spencer, 1966), and later, a tenth was added (Rankin et al., 2009). In the above studies, seven criteria were met: decreased response to repeated stimuli, sensory recovery, dependence on stimulus frequency, dependence on stimulus intensity, stimulus specificity, and dishabituation (Boisseau et al., 2016; Vogel and Dussutour, 2016) or long-term habituation (Boussard et al., 2019). This learning memory is found to be closely related to the cellular uptake and retention of stimulus chemical substances given during learning (Boussard et al., 2019), but it is not known how exactly the chemicals from the environment act on the internal system of the plasmodium, nor how the information is stored and how they cause changes in the behavior of plasmodium.

This study focused on the transport tube network formed in plasmodium. The transport tubes of plasmodium adaptively change in thickness in response to the flow rate of the cytoplasm (Tero et al., 2007). The thickening follows nonlinear dynamics and then generates bipolar states, such as thick or thin; this property is analogous to the phenomenon of neuronal firing. Furthermore, it was shown by a combination of experimental analysis and mathematical modeling that information applied locally can be retained in the spatial gradient of transport tube thickness, and it was suggested that a single transport tube itself could have a memory function (Kramar and Alim, 2021). It is expected that networks composed of transport tubes with such properties will provide plasmodium with more advanced information processing functions beyond that of a mere single cell.

Inspired by the habituation experiments of Boisseau et al. (2016) on plasmodium , this study examined the relationship of transport tube network morphology to the learning process and memory of habituation, and we discuss how memory information is read out and output to plasmodium behavior.

## 2 Materials and method

### 2.1 Preparation of organism for experiments, plasmodium of *Physarum polycephalum*

Prior to the following procedure, plasmodia of *P. polycephalum* are cultured on 1.5 w/v% agar medium (FUJIFILM Wako pure chemical industries), fed oat flakes (Premium Pure Oats, Nippon food manufacture), and incubated for approximately 1 day at 25°C in the dark.

### 2.2 Experimental setup

**FIGURE 1:**
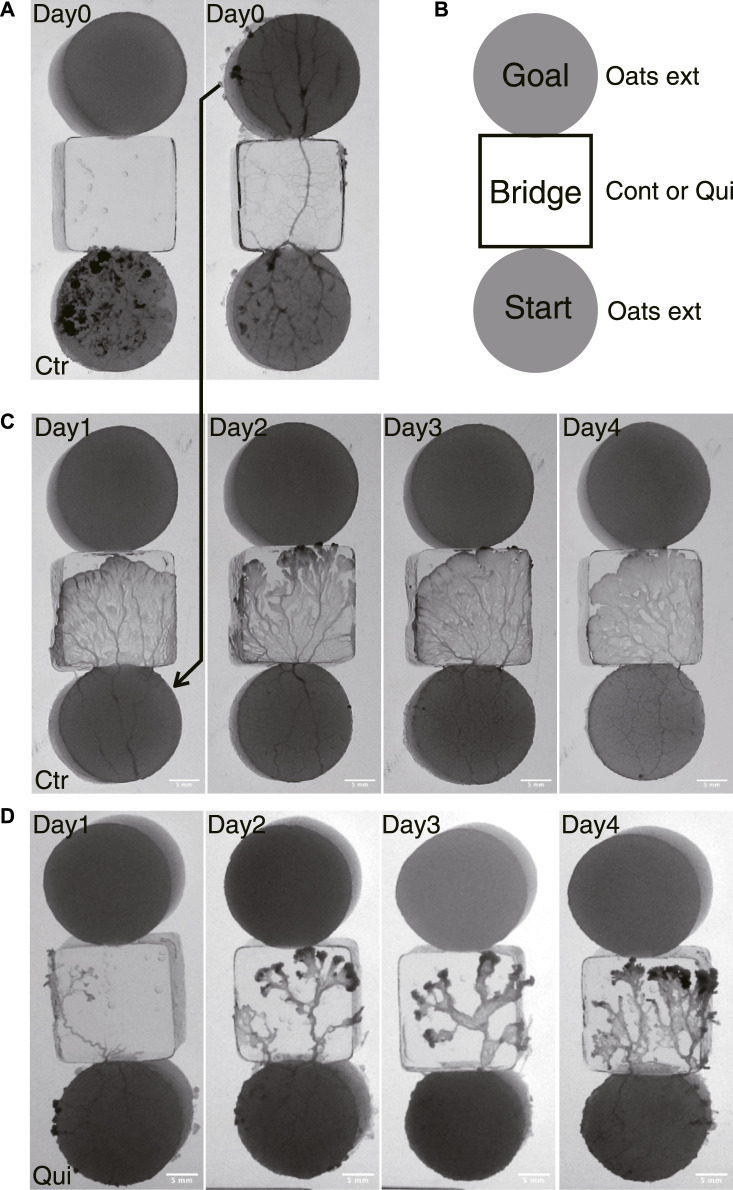
Experimental setup and typical results of Days 1–4. **(A)** An example of an experimental setup on Day 0. At *t* = 0 min, the plasmodium spread only on the start area and then begin to cross the bridge (left panel). At *t* = 905 min, the plasmodium spread toward the goal and finished crossing the bridge (right panel). The sample is a control sample. **(B)** Illustration of experimental setup. Start and goal blocks are a 1.0 w/v % agar plate containing 10 w/v %oat-flak-grind extract. The bridge is a 1.0 w/v % agar plate containing 0.5 mM Quinine for test bridges (Qui) and without chemicals for control bridges (Ctr). **(C, D)** Typical results of control and Quinine experiments on Days 1–4, respectively. The images represent the ones at the time *t*_*e*_ when the plasmodia reached the upper edge of the bridge block, which is in contact with the goal block. See also Supplementary Movie S1 (control) and Supplementary Movie S2 (Quinine) for descriptions of how the plasmodia progress across the bridges to reach the goal blocks. **(C)** Control: *t*_*e*_ = 110, 80,120,110 min. **(D)** Quinine: *t*_*e*_ = 285, 280, 210, 150 min.

The basic experimental setup consists of a start block, bridge block, and goal block, as shown in Figure 1. The start and goal blocks are food blocks made of 1.0 w/v% agar containing 10 g w/v% oat-flake-grind extract (Takamatsu et al., 2009). They are circular in shape, 18 mm in diameter and 3–4 mm thick. The bridge block is made of 1.0 w/v% agar with or without containing 0.5 mM Quinine hydrochloride dihydrate (FUJIFILM Wako pure chemical industries), which are used for Quinine experiments or control experiments, respectively. Quinine was used to inhibit the plasmodium from crossing bridges for habituation training in the same manner as in the experiments of Boisseau et al. (2016). Quinine is an alkaloid and an aversive chemical substance for the plasmodium. It was reported that application of this substance slowed the flow of cytoplasm (Kamiya, 1959), and temporarily inhibited the progress of the plasmodium on agar medium containing this substance (Takagi et al., 2007). The bridge blocks are rectangular, 18 × 18 mm in size, and 3–4 mm thick. The bridge blocks are placed between the start and goal, as shown in Figure 1B.

Thirty setups shown in Figure 1B (almost half of them were for Quinine experiments, while the rest were for control experiments) were placed on two large plastic plates (square bio assay dish, 245 × 245 mm, Corning Inc.) for a single experiment set. To avoid systematic effects, Quinine and control setups were alternately placed on the large plates. In total, eight sets of experiments were performed on different days in different seasons (October and November 2019; July, August, October, and November 2020; and February and March 2021) in Shinjuku, Tokyo. The samples were illuminated with transmitted light by yellow-LED illumination plate (around 580 nm; LM8C 311X455-35Y AItec) to avoid light and thermal response of plasmodia (Ueda et al., 1988). The experiments were performed in a thermostatic and humidistatic chamber at 25 ± 0.3°C, with a relative humidity of 85% ± 3% (PR2KT; ESPEC Corp, Japan).

**FIGURE 2:**
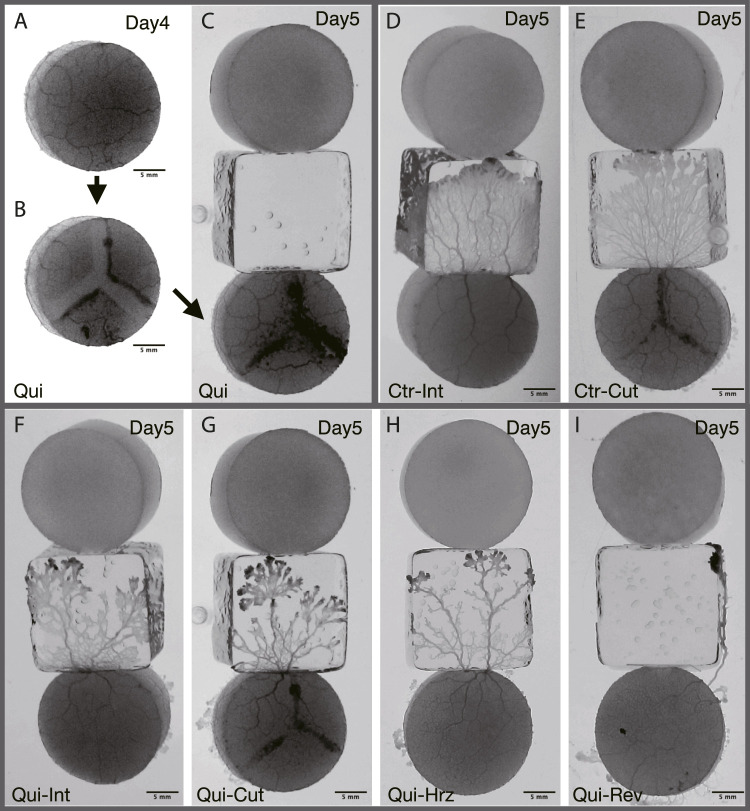
Typical results of Day 5. **(A–C)** Preparation of cutoff network. The same sample as that of the panel **(G) (A)** Plasmodium covering goal on Day 4 is used as the start sample on Day 5. *t* = −230 min in stage time on Day 5 (see Figure 3 for definition of time). **(B)** Before the Day 5 experiment, the tube network of **(A)** is cutoff in a Y-shape. *t* = −220 min. **(C)** Once the cutoff wound has healed, the sample is placed at the starting position on Day 5. *t* = −40 min. **(D, E)** Typical result of Control. See also Supplementary Movie S5. **(F–I)** Typical result of Quinine. See also Supplementary Movie S6. **(D)** Intact. *t* = 105 min. **(E)** Cutoff. *t* = 85 min. **(F)** Intact. *t* = 130 min. **(G)** Cutoff. *t* = 185 min. **(H)** Horizontal: Horizontally placed at the starting position. *t* = 155 min. **(I)** Reverse: Reversely placed at the starting position. *t* = 165 min. All results presented here are from experiments conducted on the same day. The images in **(D–I)** represent the ones at the time *t*_*e*_ when the plasmodia reached the upper edge of the bridge block, which is in contact with the goal block.

**FIGURE 3:**
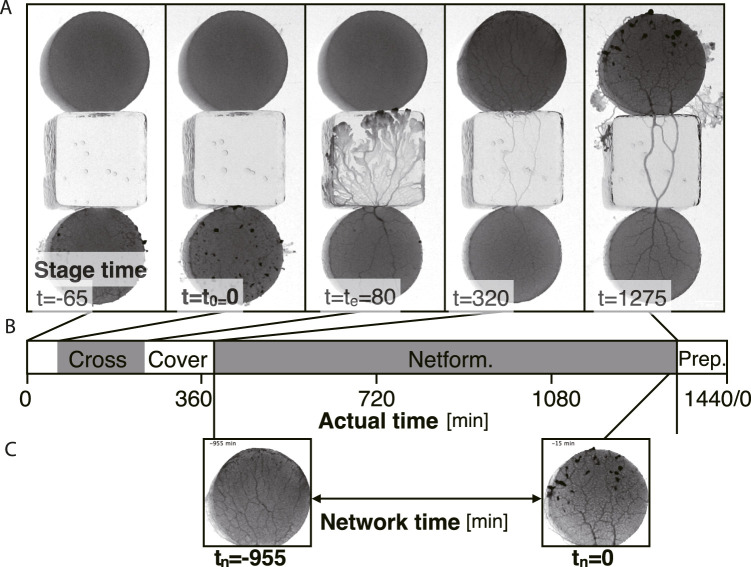
Definition of the times in a day. Time units are in minutes. **(A)** Examples of each stage at time *t*. **(B)** Time chart of each stage indicated using actual time in a day. At actual time 0 min (*t* = −65 min in stage time in this example), the start block is placed. Crossing: the plasmodium begins to cross the bridge at stage time *t* = *t*_0_ = 0 and finishes at stage time *t* = *t*_*e*_. Covering: the goal block is being covered with the plasmodium (*t* = 80–320). Network formation (Netform.): The transport tube network at the goal is formed (*t* = 320–1275). Preparation (Prep.): Preparation period for the next day’s experiment. **(C)** Network time *t*_*n*_. The time *t*_*n*_ = 0 is defined as the last time of the network formation phase. *t*_*n*_ is defined backward from *t*_*n*_ = 0. The image examples are taken from the same sample of Figure 1C Day 2.

The transmitted light images were recorded with a digital camera (body: EOS RP; lens: EF24 mm F2.8 IS USM, Canon Inc.) at 5 min intervals for approximately 24 h. Image resolutions were 0.06–0.08 mm/pixels. For the analysis of thickness oscillations, the images were captured at 5 s intervals for 10 min. The original raw images were converted linearly to 8-bit gray scale images using image processing software [Photoshop, Adobe Inc; Fiji (Schindelin et al., 2012)]. The images in the figures have been linearly adjusted in brightness and contrast according to their purpose, so that any description can be easily understood. In Figure 1A, Figure 3A, and Figure 9, the treatment focuses on the entire plasmodium on the three blocks. In Figures 1C, D, Figures 2D–I, the treatment focuses on the plasmodium on the bridge. In Figures 2A, B, Figure 3C, Figure 6 and Figure 7, the treatment focuses on the network formed by the plasmodium on the goal block.

Eight datasets were used for behavior analysis, including measuring area and speed. Six datasets were used for network analysis. Three datasets were used for connection analysis. One dataset was used for thickness oscillation analysis.

### 2.3 Procedure

A single set of experiments lasted for 6 days, from Day 0 to Day 5. For these experiments, plasmodia are cut from the fan-shaped frontal part of the cultured plasmodium (§2.1) so that the total amount is 0.085 g. As the wounds of the freshly collected plasmodia are not sufficiently healed, the Day 0 procedure is performed before the test experiments of Day 1–5 are begun. The fresh plasmodia are transferred to the start block on Day 0 (Figure 1A). The plasmodium begins to spread on the bridge block, then reaches the goal block, and finally covers it. The goal block covered by the plasmodium is detached from this setup, and then used for Day 1’s experiment (Figure 1C). On Day 1, the plasmodium at start position begins to spread on the new test bridge, then reaches and covers the new goal. These procedures are repeated until Day 4.

On Day 5, the goal plasmodia of Day 4 are divided into 4 groups named intact, cutoff, horizontal, and reverse, respectively as shown in Figure 2. The first group (intact) of the goal plasmodia are placed intact at the start of the Day 5 experiment, the same as the experiments of Days 1–4 (Figures 2D, F). The second group (cutoff) of the goal plasmodia are placed at the start after being cut in a Y-shape so that the transport tube network becomes disconnected (Figures 2B, C, G). In this procedure, the plasmodium are scratched 2–3 mm wide by gently sliding a spatula over the agar surface. Care is taken not to damage the agar surface. This procedure divides the single plasmodium into three parts as shown in Figure 2B. Note that the block are placed at starting position after their wound has healed and the three separate parts become a single body. The third and fourth groups (horizontal and reverse) of the goal plasmodia of Day 4 are placed after rotating their orientations by 90^*o*^ and 180^*o*^ from the original direction at the start positions of Day 5, respectively (Figures 2H, I).

### 2.4 Definition of times in a day

The procedure of placing the start block of Day *N* is performed at almost the same time in 1 day, which is defined as time 0 in actual time (Figure 3). Then, the experiment day finishes at actual time = 1440 min, including preparation time for the next-day experiment (Figure 3B).

Two different time definitions are introduced: stage time (Figures 3A, B) and network time (Figure 3C). The stage time *t* = *t*_0_ = 0 is defined when the plasmodium on the start block begins to cross the test bridge (Figure 3A). Then, at time *t*_*e*_, the plasmodium reaches the upper edge of the bridge block, which is in contact with the goal block. The plasmodium then enters the goal block followed by the covering state. The goal block has finished being covered by the plasmodium at *t* = 320 in this example. Through this event, the transport tube network is formed on the goal block. This network formation is observed until the time for preparation of the next day’s experiment begins, which is defined as network time *t*_*n*_ = 0 (Figure 3C). Therefore the value of the network time *t*_*n*_ is negative during covering state, whose definitions are used in Figures 6A–H and Figures 7A–H.

### 2.5 Estimation of area on the bridge

Areas of plasmodium on the bridges were estimated using Fiji (Schindelin et al., 2012). The background image, which is the image at *t*_0_ (second part of Figure 3A) was subtracted from the image at *t*_*e*_ (third part of Figure 3A; Supplementary Figure S1A). Only the part of plasmodium on the bridge was extracted by thresholding the image (Supplementary Figure S1B) so that it is binarized (Supplementary Figure S1C). Then, the pixels were counted.

### 2.6 Estimation of speed on the bridge

The speed of the plasmodium on the bridge was estimated as [bridge length (mm)]/[*t*_*e*_ − *t*_0_ (min)]. The speed of progression strongly depends on the environment of the day (wind exposure in the thermostatic and humidistatic chamber, season, and parentage of the plasmodium). To compensate for such effects, the speed data were normalized with the mean speed of the samples of a control experiment performed on the same day.

### 2.7 Characterization of tube network at goal

The tube networks at goals were characterized by two-dimensional Fourier transform analysis that captures the spatio-periodic structure. The goal image at time *t*_*n*_ was cropped into a square for Fast Fourier transform (FFT) analysis using the Fiji plugin (Supplementary Figure S2A), and then the power spectrum was obtained (Supplementary Figure S2B), which includes direction angles and power values. The angle values were weighted by power value, and then the distribution of tube orientation was obtained (Supplementary Figure S2C). Note that the 180^o^ − 360^o^ data were not included in the analysis because the spectrum is point symmetric. Finally, the major orientation angles of transport tubes in a goal network at time *t*_*n*_ were estimated as the mode of the distribution, as shown Supplementary Figure S2C, which was obtained by density estimation using the R software (R Core Team, 2023). Note that the estimated angle values are orthogonal to the orientation direction of the tube, as shown in Supplementary Figure S2D.

### 2.8 Characterization of daily change of network morphology

Characteristic angles of the day were estimated from the time course of the orientation angles. The characteristic angles were plotted in terms of daily change. These plots were manually classified from Type 0 to Type 4 with the conditions blindfolded. See §3.1.3 for details.

### 2.9 Analysis of thickness oscillation

The thickness oscillations of plasmodia on the start block during the 10 min before they begin to cross the bridge were analyzed (Supplementary Figure S3). The area of 320 × 320 pixels (about 20 × 20 mm^2^) was divided into smaller areas of 10 × 10 pixels, and then the transmitted light intensity of the plasmodium of this area was averaged for each smaller area to obtain the time series data, as shown in Supplementary Figure S3A. The time series data were smoothed by locally-weighted polynomial regression using the R “lowess” function to remove noise. The oscillation periods were estimated as the times between two successive peaks of this waveform. Then, the mode of the period distribution was used as the measure of period *T* of the small area, and the value was converted into angular frequency as 2*π*/*T*. Supplementary Figure S3B shows the spatial distribution of the angular frequencies. Supplementary Figure S3C shows a histogram of the angular frequency obtained from the data of Supplementary Figure S3B. Note that the data above half (more strict threshold than usual) of the Nyquist frequency (2*π* ⋅ [*f*_*n*_/2], where the Nyquist frequency *f*_*n*_ = 0.1) were omitted as noise. The median of this distribution was taken as the representative value of the oscillation frequency of the plasmodium on the start block, whose value was used in §3.4.

### 2.10 Statistical analysis

To determine the significance of the data, the normality and equal variance of the data groups were first subjected to the Shapiro and Bartlett tests, respectively. For data groups for which the normal distribution and equal variance were not rejected, the Dunnett test was applied as a multiple test, and for the other data groups, the Steel test was applied. The results of statistical analysis are summarized in Supplementary Table S1. These statistical analyses were performed using R.

## 3 Results

The experimental process is divided into two phases. The first phase is the process of learning “habituation” in Day 1–4, and second phase is the process of deletion of “learning memory”. It should be noted that the terms such as “habituation” are used here according to the findings by reports on plasmodium of *P. polycephalum* (Boisseau et al., 2016; Vogel and Dussutour, 2016; Boussard et al., 2019), not in the original psychological sense. Control and Quinine experiments were always simultaneously performed.

### 3.1 Learning process of “habituation”

The habituation experiments were performed from Days 1–4. On each day, the start block, which is the goal block of the previous day’s experiment and is covered with the plasmodium, was placed in contact with the bridge, as shown in Figures 1B–D. The plasmodium began to spread on the bridge (*t* = *t*_0_ in Figure 3A) after 10 min to several hours (Supplementary Figures S4A, B), irrespective of control or Quinine experiments. The tip portion of the plasmodium finish the bridge crossing at *t* = *t*_*e*_ (Figure 3A). It took 1–2.5 h for the control and 1.5–3.5 h for the Quinine experiments to finish the bridge crossing (Supplementary Figures S4C, D), whose details are discussed in §3.1.2. Then, the plasmodium covered the goal block over a 3–10 h period (Supplementary Figures S4E, F). The time for covering took longer with the passage of days in both the control and Quinine experiments. Finally, the tube network of plasmodium was formed over the start, bridge, and goal blocks, as shown in the final panel of Figure 3A (*t* = 1275; details are discussed in §3.3). The above behaviors can be seen in Supplementary Movie S1 for control experiments and Supplementary Movie S2 for Quinine experiments. The difference in characteristics of control and Quinine experiments appeared in the area on the bridges (§3.1.1) and speed of crossing them (§3.1.2), as Boisseau et al. (2016) reported.

#### 3.1.1 Area on the bridges

**FIGURE 4:**
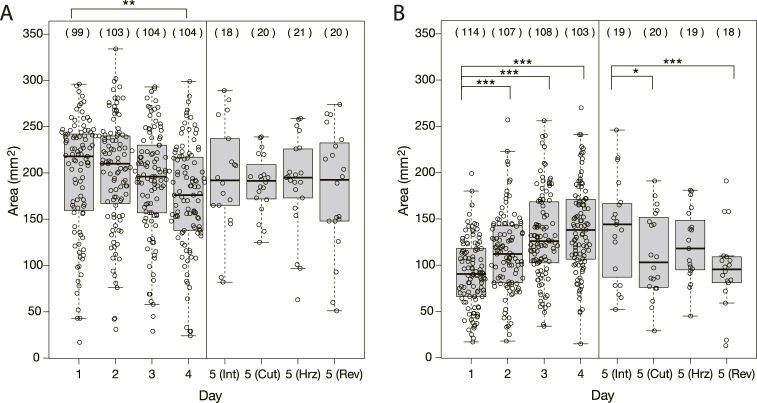
Area of plasmodia on bridges at the moment when they finished crossing bridges. **(A)** Control experiment. **(B)** Quinine experiment. The abbreviations in parentheses shown in Day 5 represent Int: intact, Cut: cutoff, Hrz: horizontally placed, Rev: reversely placed. Open circles represent data of a single sample. Thick bars in box plots are the median of each dataset; Upper and lower edges of boxes are upper and lower quantiles, respectively; Upper and lower thin bars are the largest/smallest value less/greater than upper/lower quantile plus/minus 1.5 times interquartile range, respectively; See R manual for details. The numbers in parentheses above the boxes indicate the number of data in each dataset. Asterisks represent the degree of *p*-value calculated as a result of appropriate statistical tests for each dataset as follows: * *p* < 0.1, ** *p* < 0.05, *** *p* < 0.01, and unlabeled *p* > 0.1. See Supplementary Table S1 for details. As reference data in the multiple comparison test, the Day 1 dataset was used for Days 1–4 of analysis, and the intact Day 5 dataset was used for Day 5 of analysis.

As seen in Figure 1, the shape and area of the plasmodia on the bridges over the Day 1–4 experiments were quite different between control and Quinine experiments. In the control experiments, the plasmodium spread in thin sheets over the bridge, with little daily variation in shape (Figure 1C). The area on the bridge also changed little over the days, but tended to decrease slightly on Day 4, as shown in Figure 4A. On the contrary, in the Quinine experiments, the shape (Figure 1D) and area (Figure 4B) changed markedly over the days. On Day 1, the shape is string-like and the area is very small. Each day thereafter, the shape changed to a branch-like shape with the sheet area increased (Figure 1D), and the area increased significantly (Figure 4B). These trends are consistent with the results of Boisseau et al. (2016).

#### 3.1.2 Speed of crossing the bridges

With respect to the raw data of speed of the plasmodium crossing the bridge, the results differed slightly from those of Boisseau et al. (2016). As shown in Supplementary Figure S5A, the speed in the control experiment decreased over days, whereas the results of Boisseau et al. showed no change. In the Quinine experiment, the speed seemed to increase slightly over days but not statistically significantly, whereas the results of Boisseau et al. showed a significant increase.

The difference between Boisseau et al.’s experimental setup and ours is in the preparation method of the start and goal blocks and in the concentration of Quinine in the bridge. The start and goal blocks contained 10% blended oat flakes in their setup, whereas ours contained 10 g w/v% oat-flake-grind extract because it was needed to increase the light transparency of the blocks for observation of the transport tube network. The latter is less attractive as nutrition than the former. This would affect the result of speed reduction in the control experiment. In addition to this, the tendency of reduction was not always observed: half of the eight sets of experiments showed a different tendency. As mentioned in §2.6, the speed would be affected by the environment condition of the day of experiment. Therefore, speed was evaluated using values normalized by those of the control experiment of the same date.

**FIGURE 5:**
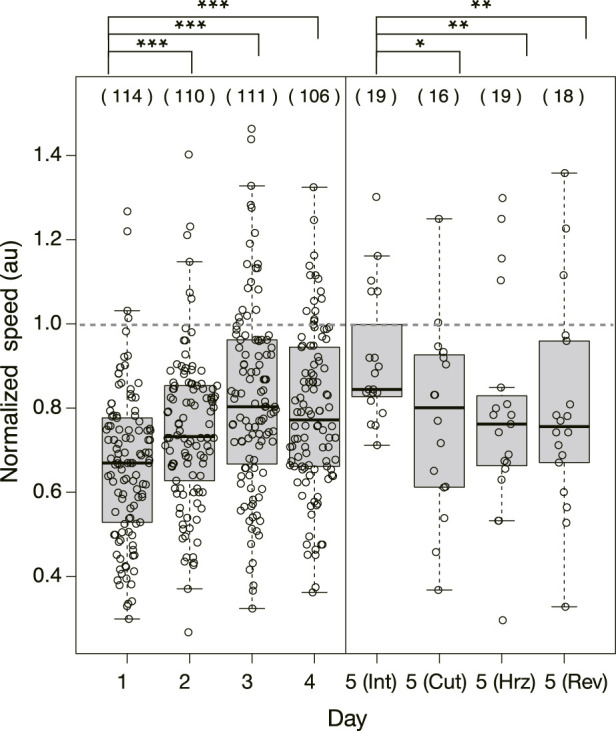
Normalized speed of plasmodium crossing the bridge in Quinine experiment. The speeds were normalized by the mean speed of samples of control experiment performed on the same day. Dashed horizontal line denotes 1.0 in normalized speed, which means the mean speed in the control experiment. Other notations are the same as those in Figure 4.

Figure 5 shows the speed of the Quinine experiment normalized with the mean speed of the control samples performed on the same day. Then, it is concluded that the normalized speed increases statistically significantly over days.

Together with the results of §3.1.1 and §3.1.2 and compared with the results of Boisseau et al., it can be concluded that the plasmodium learnt to overcome the danger of the bridge under repeated experiences, thus learning of “habituation” was established.

#### 3.1.3 Network formation during “learning” process

**FIGURE 6:**
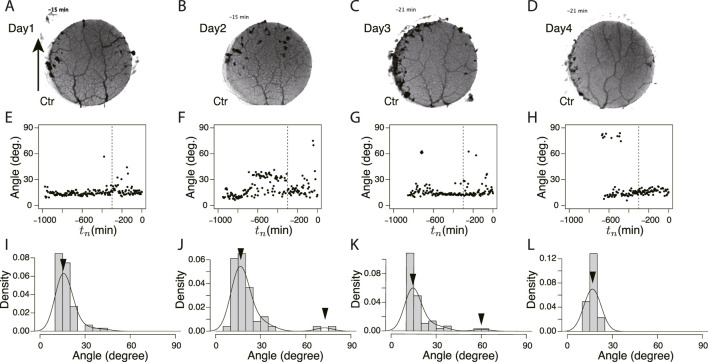
Orientation angle of transport tubes in control experiment. **(A–D)** Examples of final tube network morphology. The arrows indicate the direction in which the tip portion of the plasmodium moves. **(E–H)** Time course of major orientation angle of tube network. The angle is from the direction of the arrow shown in Figure **(A)**. The vertical dashed line is at *t*_*n*_ = −300. **(I–L)** Distribution of final major orientation angle taken from *t*_*n*_ = −300 to *t*_*n*_ = 0. The curve is a density plot in which peak angles (denoted by arrowheads) are estimated as characteristic angles of the day. These examples of the peak angles and their density values were used for Figure 8B. **(A, E, I)** Day 1, **(B, F, J)** Day 2, **(C, G, K)** Day 3, **(D, H, L)** Day 4.

**FIGURE 7:**
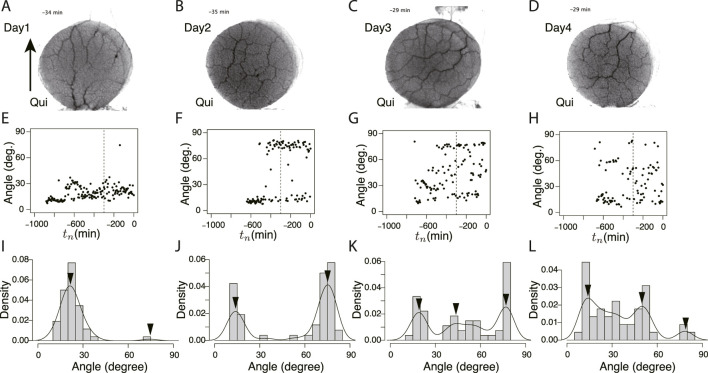
Orientation angle of transport tubes in Quinine experiment. **(A–D)** Examples of final tube network morphology. **(E–H)** Time course of major orientation angle of tube network. **(I–L)** Distribution of final major orientation angle taken from *t*_*n*_ = −300 − 0. These examples of peak angles and their density values are used for Figure 8E. **(A, E, I)** Day 1, **(B, F, J)** Day 2, **(C, G, K)** Day 3, **(D, H, L)** Day 4. Other notations are the same as those in Figure 6.

To study the state of the learning memory in the plasmodium, we focused on the network of transport tubes that form over the goal block. Figure 6 and Figure 7 show the typical results of network formation on the goal blocks after crossing control and Quinine bridges, respectively. In the control experiments, the branching network (tree) was formed, and there was little day-to-day change in the network morphology (Figures 6A–D). On the contrary, in the Quinine experiments, the network morphology changed from a tree structure to a mesh structure over the course of days (Figures 7A–D).

These typical images of the networks were obtained from the final states of the network formation process, which lasts from the covering stage to the end of the observation of the day (Supplementary Movie S3 for control experiment and Supplementary Movie S4 for Quinine experiment). To quantify the process of network formation, the orientation angles of transport tubes were examined. Figures 6E–H and Figures 7E–H show the time course of the orientation angles. For example, in Figures 6E, G, H (control, Day 1, 3, and 4), and Figure 7E (Quinine, Day 1), the orientation angles of tubes were always approximately parallel (approximately 10–30^*o*^) to a direction denoted by arrows in Figures 6A, 7B, which is the direction of the plasmodium advancing during network formation. This characterizes the tree network. Meanwhile, in Figures 7F–H (Quinine, Day 2–4), the orientation angles were initially only parallel, but later, vertical angles (about 70–80^*o*^) were added to the end. This final state characterizes the mesh network. This correspondence between orientation angle and network formation was later used to automatically determine the classification of network shapes for a large number of samples.

The angle data from *t*_*n*_ = −300 to *t*_*n*_ = 0 of Figures 6E–H and Figure 7E–H were used to determine the final states. Figures 6I–L and Figures 7I–L show the distributions of the above data. The peak angles obtained from the distribution were used as the characteristic angle of the day for the following statistical analysis of the daily change.

**FIGURE 8:**
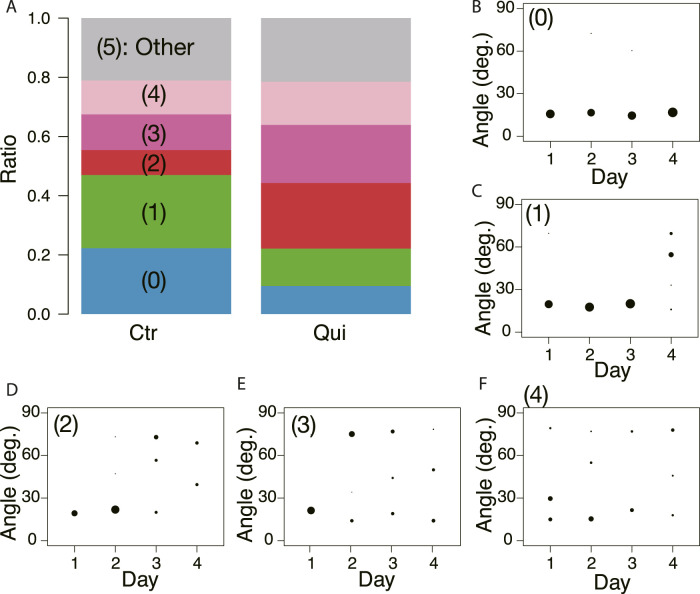
Daily change of orientation angles of tubes in the final transport networks. **(A)** Classification of daily change is shown as a ratio to the number of observations. Numbers in parentheses indicate type number. The numbers of observation data are 83 and 79 for the control and Quinine experiments, respectively. **(B)** Type 0: major tube orientations are always parallel to the direction in which the tip of the plasmodium was advancing (angle $<45^{o}$ ). **(C)** Type 1: tube orientation changes to multidirection on Day 4. **(D)** Type 2: tube orientation changes to multidirection on Day 3. **(E)** Type 3: tube orientation changes to multidirection on Day 2. **(F)** Type 4: tube orientation is always multidirection. Type 5 is classified as other changes.

Figure 8 shows the classification of network formation according to the daily change in characteristic angle. The typical daily changes are shown in Figures 8B–F. In Figure 8B, the major tube orientations are always parallel to the direction in which the tip of the plasmodium was advancing (Type 0). This indicates that the network is always a tree. In Figures 8C–F, the orientation of tubes changes to multidirectional on Day 4–1, respectively (named type 1–4, respectively). Here, the multidirectional tubes indicate that the network is mesh type. As shown in Figure 8A, Type 0 and 1 are the majority in the control experiment, while Type 2–4 are the majority in the Quinine experiment. This quantitative analysis supports the observations of the typical case described at the beginning of this section.

### 3.2 Deletion of “learning memory”

As shown in §3.1.3, it was confirmed that the network morphology changed during the learning process of “habituation” in the Quinine experiment. This indicates that the network morphology relates to the state of the learning memory of the plasmodium. To verify this hypothesis, Day 5 experiments were performed to see whether memory status changes when the network of the start blocks is artificially disrupted or when the start blocks are placed at different orientations. Figure 2 shows a typical view of plasmodium spreading on bridges after these manipulations. In the control experiment, there is not much change in the area after cutting the network (Figure 2E; Supplementary Movie S5) compared to the intact case (Figure 2D). On the contrary, in the Quinine experiment, after cutting or changing direction of the network, the area is smaller than in the intact case (Figures 2F–I; Supplementary Movie S6). These results can be statistically confirmed through the quantitative analysis of the area, as shown in Figure 4. In addition, in the Quinine experiment, when the network is manipulated, the speed of crossing the bridge becomes smaller than for the intact case, as shown in Figure 5. Taken together, these results suggest that there is a strong relationship between learning memory and the maintenance of network morphology.

### 3.3 Relation of network formation with the types of connections between the start and goal blocks

**FIGURE 9:**
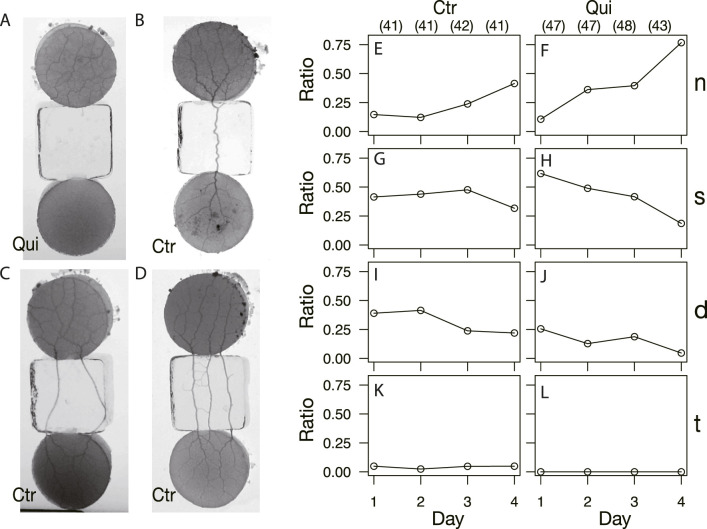
Types of start and goal connections after network formation. **(A, E, F)** No-connection (*n*). **(B, G, H)** Single tube (*s*). **(C, I, J)** Double tube (*d*). **(D, K, L)** Triple tube (*t*); the connections with more than three tubes are also included in this category. **(A–D)** Images are those of almost final phase of network formation. **(A)** Quinine experiment on Day 3. **(B, C)** Control experiment on Day 3. **(D)** Control experiment on Day 1. **(E–L)** Daily change of observation ratio of each connection type. The denominator of the ratio is the number of data in the same conditions on the same day, which are shown in parentheses placed at the top of Figures **(E)** and **(F)**.

As shown in §3.1.3, the network formation differed between the control and Quinine experiments. To investigate how these different networks are formed, the type of tube connection between the start and goal blocks was examined (Figure 9). There are four types of connections: there is no connection between the start block and the goal block, as shown in Figure 9A (Type *n*); the number of connection tubes is single, double, and triple, as shown in Figures 9B–D (Type *s*, *d*, and *t*), respectively. Note that type *t* also categorizes the case where the number of connection tubes is greater than three. The observation ratio of Type *n* increases with the passage of days, regardless of whether the experiment is control or Quinine (Figures 9E, F). It should be noted that the values grow much larger in the Quinine experiments, especially on Days 2–4. On the contrary, the ratios of Types *s* and *d* significantly decrease with the days in the Quinine experiments, while they are almost constant in the control experiments (Figures 9G–J). Finally, on Day 4, the case that the start and goal blocks are strongly connected is dominant in the control experiments, whereas the case of no-connection is dominant in the Quinine experiments. This suggests that the strength of connection affects network formation on the goal block.

**FIGURE 10:**
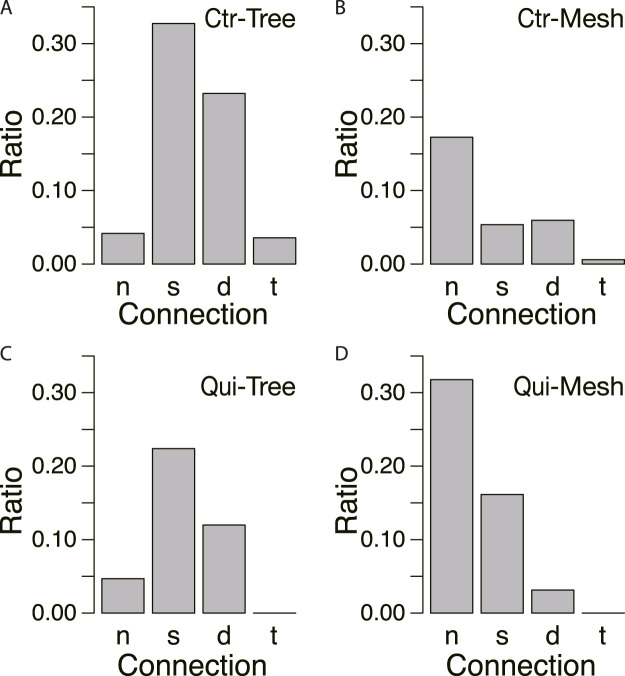
Relation between connections and network morphology. **(A, B)** Control experiment. *N* = 165. **(C, D)** Quinine experiment. *N* = 185. Denominator of ratio is the number of data in the same conditions. The meanings of symbols of connection types are as follows: no-connection (*n*), single tube (*s*), double tube (*d*), triple tube (*t*). The data from Days 1–4 are combined. Network morphologies are tree in A and C and mesh in B and D, which were determined based on whether the characteristic angle is small (tree network) or multiple (mesh network) from the data of Figure 8.

To confirm this assumption, the ratio of connecting types was examined for each type of network formed in the goal, as shown in Figure 10. In the control, tree networks are very common, and the results are concentrated in the cases of strong connections (Type *s*, *d*, and *t*; Figure 10A). A small number of mesh networks can also be observed in the control experiments, but they are more common in the absence of connections (Type *n*; Figure 10B). In contrast, in the Quinine experiments, Type *n* connection is the most frequently observed in the mesh networks, as shown in Figure 10D.

To summarize the above results, a tree-type network is formed when start and goal blocks are connected through transport tubes. Conversely, if plasmodia on both blocks are separated by bridges containing repellent materials, the network will be formed only within the goal block, resulting in a mesh-type network. In the control experiment environment, the plasmodium on the start and goal blocks remained strongly connected via tubes developed on the bridges (Figures 9B–D). In this dumbbell-shaped morphology of the plasmodium, the cytoplasm of the plasmodium frequently moves back and forth between the start and goal, autocatalytically strengthening the connection between the two portions (Nakagaki et al., 2000b; Takamatsu et al., 2000). Thus, the reciprocal flow of protoplasm from the start to goal direction (arrow direction shown in Figure 6A) may have led to the development of transport tubes parallel to this direction, resulting in the formation of a tree-shaped network. Meanwhile, in the Quinine experiment environment, the start and goal blocks became separated as the number of experimental days passed, and the networks were formed only on the goal block (Figure 9A). Of course, plasmodia sometimes remain in the start block as well; however, we will ignore them here because they will not be used in the next day’s experiment. The reason for the division of the two blocks is supposed to be due to the tendency of the plasmodium to escape from aversive chemicals contained in the bridge blocks. Within a limited area on the goal block, no cross-sectional cytoplasmic flow over the goal block is observed. In addition, the goal contains nutrition, on which it is known that a mesh-type network is formed (Takamatsu et al., 2009; Ito et al., 2011). Due to both above effects, a mesh network would be formed in the later days of the Quinine experiment.

### 3.4 Relation between frequency of thickness oscillation and network morphology

**FIGURE 11:**
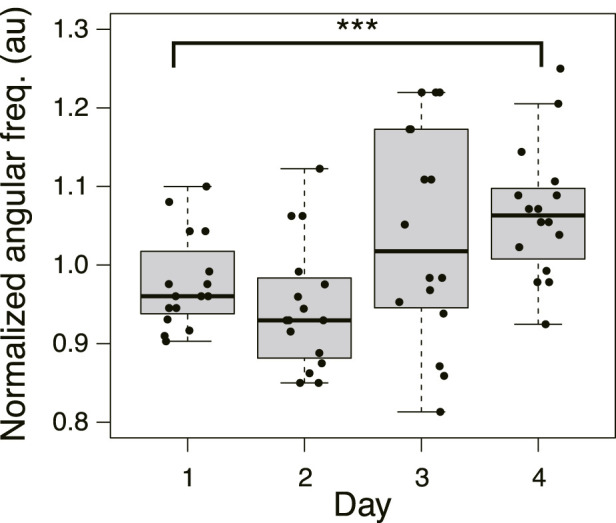
Angular frequency of thickness oscillation on start block in Quinine experiment. *N* = 16 for all daily data. Data are normalized by the median of the control data on the same day. See Supplementary Figure S6 for details. Asterisks represent the degree of *p*-value calculated as a result of appropriate statistical tests for each data set as follows: *** *p* < 0.01, and unlabeled *p* > 0.1. As reference data in the multiple comparison test, the Day 1 dataset was used. See Supplementary Table S1 for details.

Based on the above results, after crossing a Quinine bridge, a tree network is formed in the goal block on Day 1 or 2 in most cases, and a mesh network is formed on Day 3 or 4. Meanwhile, the area of plasmodium on a bridge increases with the passage of days, and the relative crossing speed also increases. To investigate how these different morphologies of networks affect the bridge-crossing behavior of the plasmodium, the thickness oscillation of the plasmodium at the start blocks was investigated. Figure 11 shows the oscillation frequency in the Quinine experiments normalized by the median of the control data on the same day (see Supplementary Figure S6 for the original data). The frequencies are lower on Days 1 and 2 compared to the control data (denoted by 1.0 of the normalized angular frequency) and then become higher on Days 3 and 4. The results suggest that higher frequency of thickness oscillation assists the plasmodium to pass through the Quinine bridge, increasing its speed and spreading area. To confirm this hypothesis, the relations between oscillation frequency and the quantitative indices of behavior of the plasmodium on the bridges, such as area and speed, were analyzed. However, it was difficult to find direct correlations between the frequency and the behavioral indices (Supplementary Figure S7), which might be due to the wide dispersion of the data.

**FIGURE 12:**
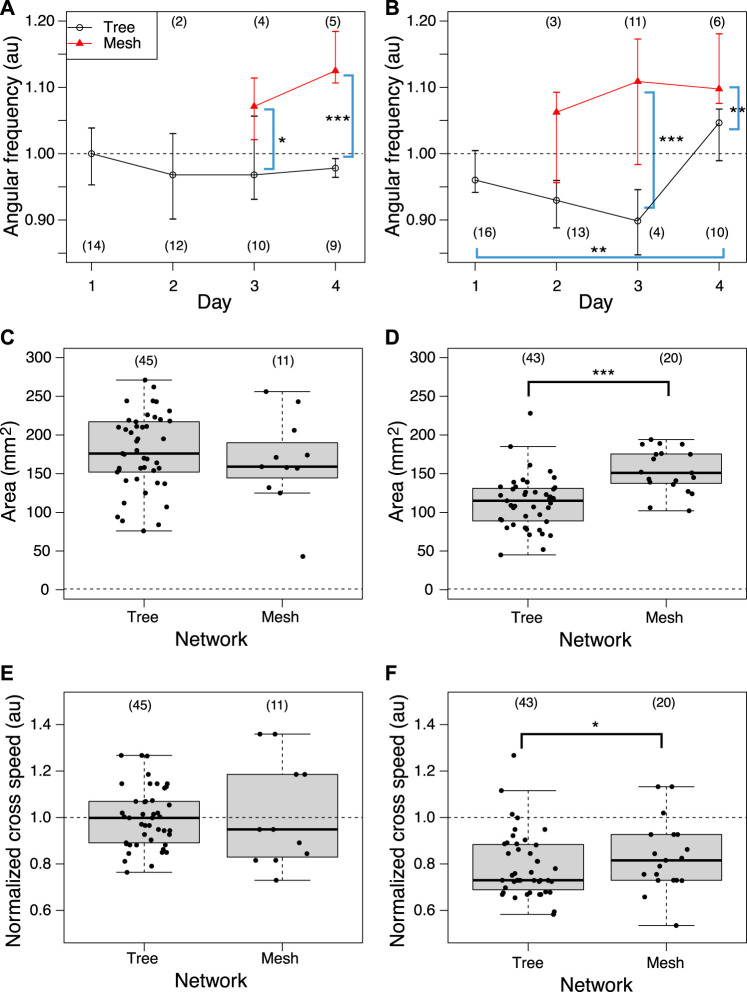
Relation between network morphology and quantitative indices of habituation behavior. **(A, B)** Angular frequency of thickness oscillation at start block. Angular frequencies are normalized by the median of the control data on the same day. Network morphologies are classified using those of the previous day’s goal. **(C, D)** Area. **(E, F)** Crossing speed. **(A, C, E)** Control experiment. **(B, D, F)** Quinine experiment. Numbers in parentheses indicate number of data in each dataset. Asterisks represent the degree of *p*-value calculated as a result of appropriate statistical tests for each dataset as follows: *** *p* < 0.01, and unlabeled *p* > 0.1. Steel tests (one sided; greater) were applied to test daily changes using the Day 1 dataset as reference data (or earlier date if the number of data is less than 3) for multiple comparison in Figures **(A)** and **(B)**. Mann-Whitney tests (one sided) were applied to test for differences by network morphology. See Supplementary Table S1 for details.

Then, the oscillation frequency data were analyzed separately for each network morphology to clarify what causes the oscillation frequency to rise on the later days, as shown in Figures 12A, B. Regardless of whether it was a control or Quinine experiment, the frequency of plasmodium oscillations in the mesh networks was always higher than in the tree networks. This indicates that the network morphology is a factor in controlling the oscillation frequency.

Similarly, the effect of network morphology on behavioral indices was investigated, as shown in Figures 12C–F. Both area and speed were found to be greater when the plasmodium placed at the start formed a mesh network than a tree network in the Quinine experiment, while there was no significant difference in network morphology in the control experiment.

## 4 Discussion

We trained plasmodium of true slime mold *P. polycepharum* to cross a bridge containing Quinine, an aversive substance, in accordance with the procedure of Boisseau et al. (2016). As shown in the results of §3.1.1 and §3.1.2, we confirmed that “habituation learning” was established after 4 days of training in Quinine experiment, which agreed with the results of Boisseau et al. (2016), who conducted the first habituation experiment with plasmodium. The experiments performed by Boussard et al. (2019) showed that the uptake and retention of the stimulants given during the learning process are deeply involved in the mechanism of habituation. However, it is unclear how the uptake chemicals act on the plasmodia themselves to bring the memory function and how this memory results in changes in the behavior of the plasmodia.

We hypothesized that the key to the answer lies in the transport tube network of plasmodium, and we quantitatively examined the network morphology formed in the goal, as shown in §3.1.3. In the Quinine experiment, most formed a tree network in the goal on the first day, and their morphology changed to a mesh network over the course of daily repetitive stimulation (Figure 7; Figure 8). If the retention of the aversive chemical taken into the cell is the only factor important for memory retention, then disrupting the network should not result in a change in habituation behavior (memory deletion). However, when the network formed after learning was disrupted or redirected, the behavior changed as if the memory had been deleted (§3.2). Therefore, it is reasonable to hypothesize that not only the uptake of the chemical, but also the morphology of the transport tube network would assist the habituation behavior in some manner.

**FIGURE 13:**
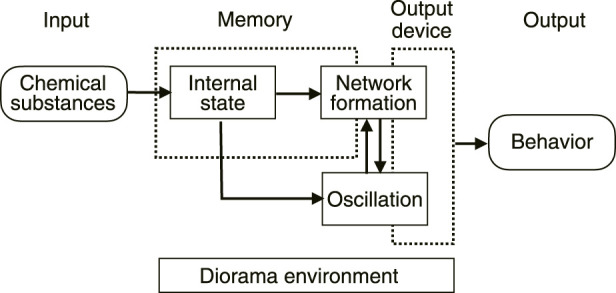
Conceptual diagram of memory in plasmodium of true slime mold.

In fact, it has already been shown that the morphology of the network depends on the chemical substances in the environment (Takamatsu et al., 2009). From this, it can be deduced that the uptake of chemicals from the environment changes the internal state of the plasmodium, namely, the physical properties of the intracellular sol and/or outer gel, which leads to a change in the network morphology. For “habituation memory”, the uptake of chemicals into the cell would be necessary to rewrite the internal state, and the change in network morphology would be necessary as a gateway to the output (Figure 13).

The learning mechanism connecting from network morphology to habituation behavior has not been elucidated. As a candidate mechanism, we expected that the thickness oscillation of plasmodium would act as a pump because of the following reports: it is known that different network morphologies produce different spatio-temporal oscillation patterns (Takamatsu et al., 2001); it has been reported that the oscillation frequency of plasmodium is high in the attractive environment and low in the aversive environment (Takahashi et al., 1997). Our experiment results confirmed that the oscillation frequency is higher when a mesh network is formed (§3.4; Figure 12B). Meanwhile, the behavioral indices of habituation, the area and speed of plasmodium spreading on the Quinine bridges, were higher when a mesh network was formed than when a tree network was formed, (Figure 12D, F). The results of these two quantitative analyses lead to the inference that the formation of the mesh network may assist the action of crossing the Quinine bridge by increasing the oscillation frequency. However, no direct evidence was found to support the hypothesis that thickness oscillations act as a pump to support the bridge cross. Further investigation is needed on this.

The following questions also remain. In the Quinine experiments, as discussed previously, the relationship between habituation behavior and network morphology was clear: the plasmodium was inactive in crossing the Quinine bridge when the network was a tree, and it was active when it was a mesh. In contrast, in the control experiment, there was little change in behavior whether the network was a tree or mesh. This does not lead to the conclusion that the morphology of the network simply causes changes in behavior. Actually, the environment for the plasmodia of the goal blocks is complicated in this experimental setup: the goal block contains a nutrient medium, while the learned plasmodium additionally uptakes an aversive chemical substance. These complex factors need to be investigated in future studies.

## 5 Conclusion

In this paper, from the memory deletion experiment, we have shown that transport tube network in addition to the retention of the chemicals plays an important role in habituation behavior in plasmodium. There would be advantages for the system where information input from the outside is not only stored internally in the form of concentration of chemical substance, but also converted into transport tube network morphology. This is because external stimuli can take various forms, such as thermal and mechanical stimuli in addition to stimuli by chemical substances. It was mentioned in the Introduction section that the transport tubes of plasmodium have neuron-like properties. Moreover, the thickness of transport tubes can be strengthened or weakened by the relationship between the oscillations of the parts (phase difference of the oscillation), resulting in the formation of different morphologies of transport tube networks. This reminds us of strengthening synapses in neurons and the formation of neural networks. Although learning functions in various single-celled organisms are widely reported, plasmodium may be the closest single-celled organism to a neural network because of the transport tube network. It would be important to study such primitive networks to hypothesize their evolution into more sophisticated neural networks.

## Data Availability

The original contributions presented in the study are included in the article/Supplementary Material, further inquiries can be directed to the corresponding author.

## References

[B1] Barron A. B. Hebets E. A. Cleland T. A. Fitzpatrick C. L. Hauber M. E. Stevens J. R. (2015). Embracing multiple definitions of learning. Trends Neurosci. 38, 405–407. 10.1016/j.tins.2015.04.008 25987443

[B2] Boisseau R. P. Vogel D. Dussutour A. (2016). Habituation in non-neural organisms: evidence from slime moulds. Proc. R. Soc. Lond. Ser. B Biol. Sci. 283, 20160446–20160447. 10.1098/rspb.2016.0446 PMC4855389 27122563

[B3] Boussard A. Delescluse J. Pérez-Escudero A. Dussutour A. (2019). Memory inception and preservation in slime moulds: the quest for a common mechanism. Philosophical Trans. R. Soc. B Biol. Sci. 374, 20180368. 10.1098/rstb.2018.0368 PMC6553583 31006372

[B4] Dussutour A. (2021). Learning in single cell organisms. Biochem. Biophysical Res. Commun. 564, 92–102. 10.1016/j.bbrc.2021.02.018 33632547

[B5] Eisenstein E. Brunder D. Blair H. (1982). Habituation and sensitization in an aneural cell: some comparative and theoretical considerations. Neurosci. Biobehav. Rev. 6, 183–194. 10.1016/0149-7634(82)90054-9 6285234

[B33] William F. D. Dee J. Hatano S. Haugli F. B. Wohlfarth-Bottermann K.-E. (Editors) (1986). “The molecular Biology of physarum polycephalum,” NATO ASI series (Berlin, Germany: Springer). 10.1007/978-1-4613-2203-0

[B6] French J. W. (1940). Trial and error learning in paramecium. J. Exp. Psychol. 26, 609–613. 10.1037/h0059015

[B7] Gunawardena J. (2022). Learning outside the brain: integrating cognitive science and systems Biology. Proc. IEEE 110, 590–612. 10.1109/jproc.2022.3162791

[B8] Hanzel T. E. Rucker W. B. (1972). Trial and error learning in paramecium: a replication. Behav. Biol. 7, 873–880. 10.1016/S0091-6773(72)80180-9 4655406

[B9] Ito M. Okamoto R. Takamatsu A. (2011). Characterization of adaptation by morphology in a planar biological network of plasmodial slime mold. J. Phys. Soc. Jpn. 80, 074801. 10.1143/jpsj.80.074801

[B10] Kamiya N. (1959). Protoplasmic streaming. Wien: Springer-Verlag. 10.1007/978-3-7091-5750-3

[B11] Kramar M. Alim K. (2021). Encoding memory in tube diameter hierarchy of living flow network. Proc. Natl. Acad. Sci. 118, e2007815118. 10.1073/pnas.2007815118 33619174 PMC7958412

[B12] Nakagaki T. (2021). Ethological dynamics in diorama environments. Available at: https://diorama-ethology.jp/eng/greeting.html. 10.3389/fcell.2023.1347957 PMC10797284 38250323

[B13] Nakagaki T. Kobayashi R. Nishiura Y. Ueda T. (2004). Obtaining multiple separate food sources: behavioural intelligence in the Physarum plasmodium. Proc. R. Soc. Lond. Ser. B Biol. Sci. 271, 2305–2310. 10.1098/rspb.2004.2856 PMC1691859 15539357

[B14] Nakagaki T. Yamada H. Tóth A. (2000a). Maze-solving by an amoeboid organism. Nature 407, 470. 10.1038/35035159 11028990

[B15] Nakagaki T. Yamada H. Ueda T. (2000b). Interaction between cell shape and contraction pattern in the Physarum plasmodium. Biophys. Chem. 84, 195–204. 10.1016/s0301-4622(00)00108-3 10852307

[B16] Rajan D. Makushok T. Kalish A. Acuna L. Bonville A. Almanza K. C. (2023). Single-cell analysis of habituation in Stentor coeruleus. Curr. Biol. 33, 241–251.e4. 10.1016/j.cub.2022.11.010 36435177 PMC9877177

[B17] Rankin C. H. Abrams T. Barry R. J. Bhatnagar S. Clayton D. F. Colombo J. (2009). Habituation revisited: an updated and revised description of the behavioral characteristics of habituation. Neurobiol. Learn. Mem. 92, 135–138. 10.1016/j.nlm.2008.09.012 18854219 PMC2754195

[B18] R Core Team (2023). R: a language and environment for statistical computing. Vienna, Austria: R Foundation for Statistical Computing.

[B19] Saigusa T. Tero A. Nakagaki T. Kuramoto Y. (2008). Amoebae anticipate periodic events. Phys. Rev. Lett. 100, 4, 018101. 10.1103/physrevlett.100.018101 18232821

[B20] Schindelin J. Arganda-Carreras I. Frise E. Kaynig V. Longair M. Pietzsch T. (2012). Fiji: an open-source platform for biological-image analysis. Nat. Methods 9, 676–682. 10.1038/nmeth.2019 22743772 PMC3855844

[B21] Takagi S. Nishiura Y. Nakagaki T. Ueda T. Ueda K.-I. (2007). Indecisive behavior of amoeba crossing an environmental barrier. Topol. Aspects Crit. Syst. Netw. 2007, 86–93. 10.1142/9789812708687_0011

[B22] Takahashi K. Takamatsu A. Hu Z. S. Tsuchiya Y. (1997). Asymmetry in the self-sustained oscillation ofPhysarum plasmodial strands. Protoplasma 197, 132–135. 10.1007/bf01279891

[B23] Takamatsu A. Fujii T. Endo I. (2000). Time delay effect in a living coupled oscillator system with the plasmodium of physarum polycephalum. Phys. Rev. Lett. 85, 2026–2029. 10.1103/physrevlett.85.2026 10970674

[B24] Takamatsu A. Gomi T. Endo T. Hirai T. Sasaki T. (2017). Energy-saving with low dimensional network in Physarum plasmodium. J. Phys. D Appl. Phys. 50, 154003. 10.1088/1361-6463/aa635a

[B25] Takamatsu A. Takaba E. Takizawa G. (2009). Environment-dependent morphology in plasmodium of true slime mold *Physarum polycephalum* and a network growth model. J. Theor. Biol. 256, 29–44. 10.1016/j.jtbi.2008.09.010 18929578

[B26] Takamatsu A. Tanaka R. Yamada H. Nakagaki T. Fujii T. Endo I. (2001). Spatiotemporal symmetry in rings of coupled biological oscillators of physarum plasmodial slime mold. Phys. Rev. Lett. 87, 078102. 10.1103/physrevlett.87.078102 11497921

[B27] Tero A. Kobayashi R. Nakagaki T. (2007). A mathematical model for adaptive transport network in path finding by true slime mold. J. Theor. Biol. 244, 553–564. 10.1016/j.jtbi.2006.07.015 17069858

[B28] Tero A. Takagi S. Saigusa T. Ito K. Bebber D. P. Fricker M. D. (2010). Rules for biologically inspired adaptive network design. Science 327, 439–442. 10.1126/science.1177894 20093467

[B29] Thompson R. F. Spencer W. A. (1966). Habituation: a model phenomenon for the study of neuronal substrates of behavior. Psychol. Rev. 73, 16–43. 10.1037/h0022681 5324565

[B30] Ueda T. Mori Y. Nakagaki T. Kobatake Y. (1988). Action spectra for superoxide generation and uv and visible light photoavoidance in plasmodia of *physarum polycephalum* . Photochem. Photobiol. 48, 705–709. 10.1111/j.1751-1097.1988.tb02884.x

[B31] Vogel D. Dussutour A. (2016). Direct transfer of learned behaviour via cell fusion in non-neural organisms. Proc. R. Soc. Lond. Ser. B Biol. Sci. 283, 20162382–20162386. 10.1098/rspb.2016.2382 PMC5204175 28003457

[B32] Watanabe S. Tero A. Takamatsu A. Nakagaki T. (2011). Traffic optimization in railroad networks using an algorithm mimicking an amoeba-like organism, Physarum plasmodium. Biosystems 105, 225–232. 10.1016/j.biosystems.2011.05.001 21620930

[B34] Wright C. S. Joshi K. Iyer-Biswas S. (2023). Cellular learning: habituation sans neurons in a unicellular organism. Curr. Biol. 33, R61–R63. 10.1016/j.cub.2022.12.008 36693308

[B35] Yi T.-M. Huang Y. Simon M. I. Doyle J. (2000). Robust perfect adaptation in bacterial chemotaxis through integral feedback control. Proc. Natl. Acad. Sci. 97, 4649–4653. 10.1073/pnas.97.9.4649 10781070 PMC18287

[B36] Zhu L. Aono M. Kim S.-J. Hara M. (2013). Amoeba-based computing for traveling salesman problem: long-term correlations between spatially separated individual cells of Physarum polycephalum. Biosystems 112, 1–10. 10.1016/j.biosystems.2013.01.008 23438635

[B37] Zhu L. Kim S.-J. Hara M. Aono M. (2018). Remarkable problem-solving ability of unicellular amoeboid organism and its mechanism. R. Soc. Open Sci. 5, 180396. 10.1098/rsos.180396 30662714 PMC6304117

