# Supplementary material for: Relation between learning process and morphology of transport tube network in plasmodium of Physarum polycephalum
Source: Front Cell Dev Biol. 2023 Nov 10;11:1249165. doi: 10.3389/fcell.2023.1249165 (PMC10667701; doi:10.3389/fcell.2023.1249165)
Supplement: Supplementary file 3 [file DataSheet1.PDF]

frontiers

Supplementary Material

RELATION BETWEEN LEARNING PROCESS AND MORPHOLOGY OF TRANSPORT TUBE NETWORK IN PLASMODIUM OF TRUE SLIME MOLD

E. Yoneoka and A. Takamatsu

1 SUPPLEMENTARY TABLE

Supplementary Table 1 can be found in a separate file.

2 SUPPLEMENTARY MOVIES

Supplementary Movies S1–6 can be found in separate files.

Movie S1. Plasmodium crossing the bridge from the start block to the goal block in control experiment. The left to right panels correspond to the observations of Days 1-4, respectively. The time is shown in the upper right corner as stage time. The unit of time is minutes. The data are the same as those of Figure 1C in the main text.

Movie S2. Plasmodium crossing the bridge from the start block to the goal block in Quinine experiment. The time is shown in the upper left corner as stage time. The unit of time is minutes. The data are the same as those of Figure 1D in the main text.

Movie S3. Network formation at the goal block in control experiment. The left to right panels correspond to the observations of Days 1-4, respectively. The time is shown in the upper left corner of each panel as network time. The unit of time is minutes. The start time of the movie is adjusted by the beginning of the covering stage. To make the network morphology easier to observe, the brightness of the images was adjusted linearly in gray scale from the original images. The data are the same as those of Figure 6 in the main text.

Movie S4. Network formation at the goal block in Quinine experiment. The data are the same as those of Figure 7 in the main text. Other notations are the same as those of Movie S3.

Movie S5. Plasmodium crossing the bridge from the start block to goal block in control experiment on Day 5. The left and right panels correspond to the data of intact and cutoff, respectively. The time is shown in the upper left corner as stage time. The unit of time is minutes. The data are the same as those of Figure 2D and E in the main text.

Movie S6. Plasmodium crossing the bridge from the start block to goal block in Quinine experiment on Day 5. The left to right panels correspond to the observations of intact, cutoff, horizontally placed, and reversely placed start blocks, respectively. The time is shown in the upper left corner as stage time. The unit of time is minutes. The data are the same as those of Figure 2F–I in the main text.

3 SUPPLEMENTARY FIGURES

Supplementary figures S1–S7 are shown below.

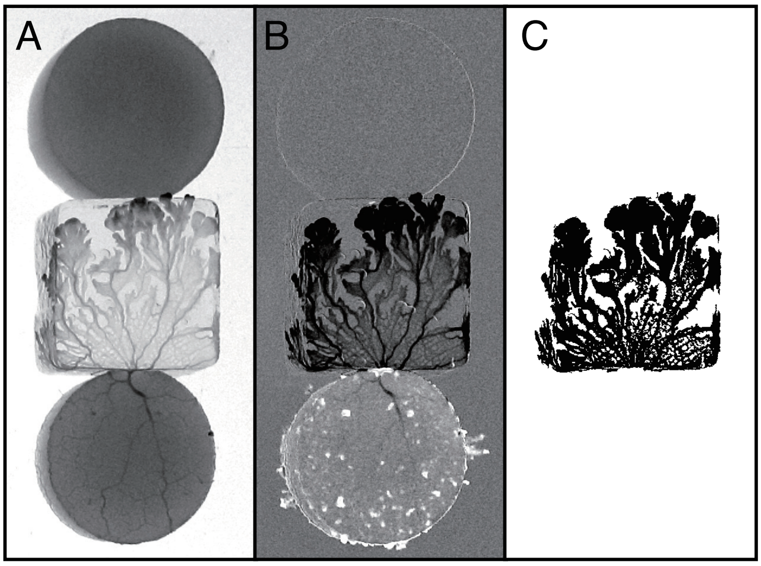

Figure S1. Estimation method of area at bridge block. (A) Gray scale image. (B) The image at time $t = 0$ is subtracted from the image A. (C) Binarized image of B with appropriate thresholding so that only the plasmodium on the bridge is extracted. The Image example is taken from the same sample as that in Figure 1C on Day 2.

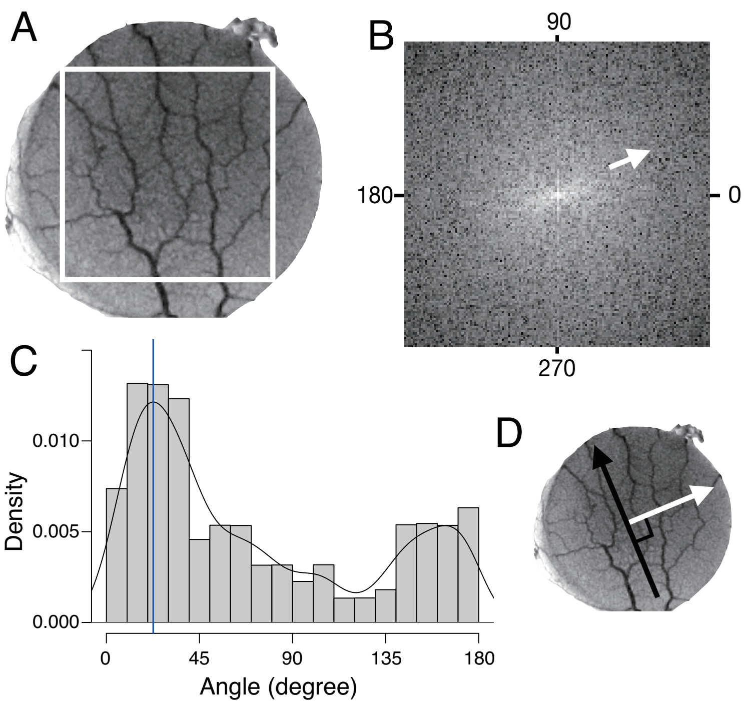

Figure S2. Estimation method of the orientation angle of the transport tubes by twodimensional fast Fourier transform (2D-FFT) analysis. (A) Tube network formed on goal. White square denotes the scope window for the analysis. (B) Power spectrum of the image A. White pixels indicate high power in the spectrum. White arrow denotes one of the orientations with high power. (C) Histogram and density plot of angle distribution. Blue vertical line denotes the mode angle estimated from the density plots (22.7^{o} in this example). (D) Relation between estimated angle by 2D-FFT (white arrow) and orientation direction of the tubes (black arrow).

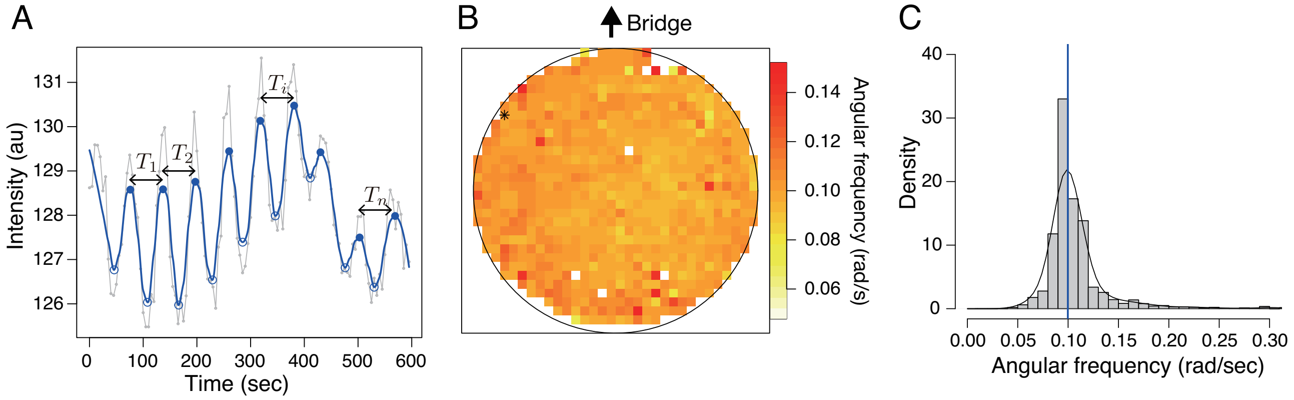

Figure S3. Thickness oscillation frequency of the plasmodium on the start block before it crossing the bridge. (A) Time course of thickness at an analysis window of 10 X 10 pixels. Blue line is obtained by smoothing the original data (gray plots and polylines). Closed blue circles are the peaks of the oscillation, and open circles are the bottoms. (B) Spatial distribution of oscillation frequency at a start block. The example of Figure A is obtained from the position denoted with an asterisk. (C) Distribution of oscillation frequency obtained from the spatial data B. Vertical blue line is the median. Black curve is the density plot.

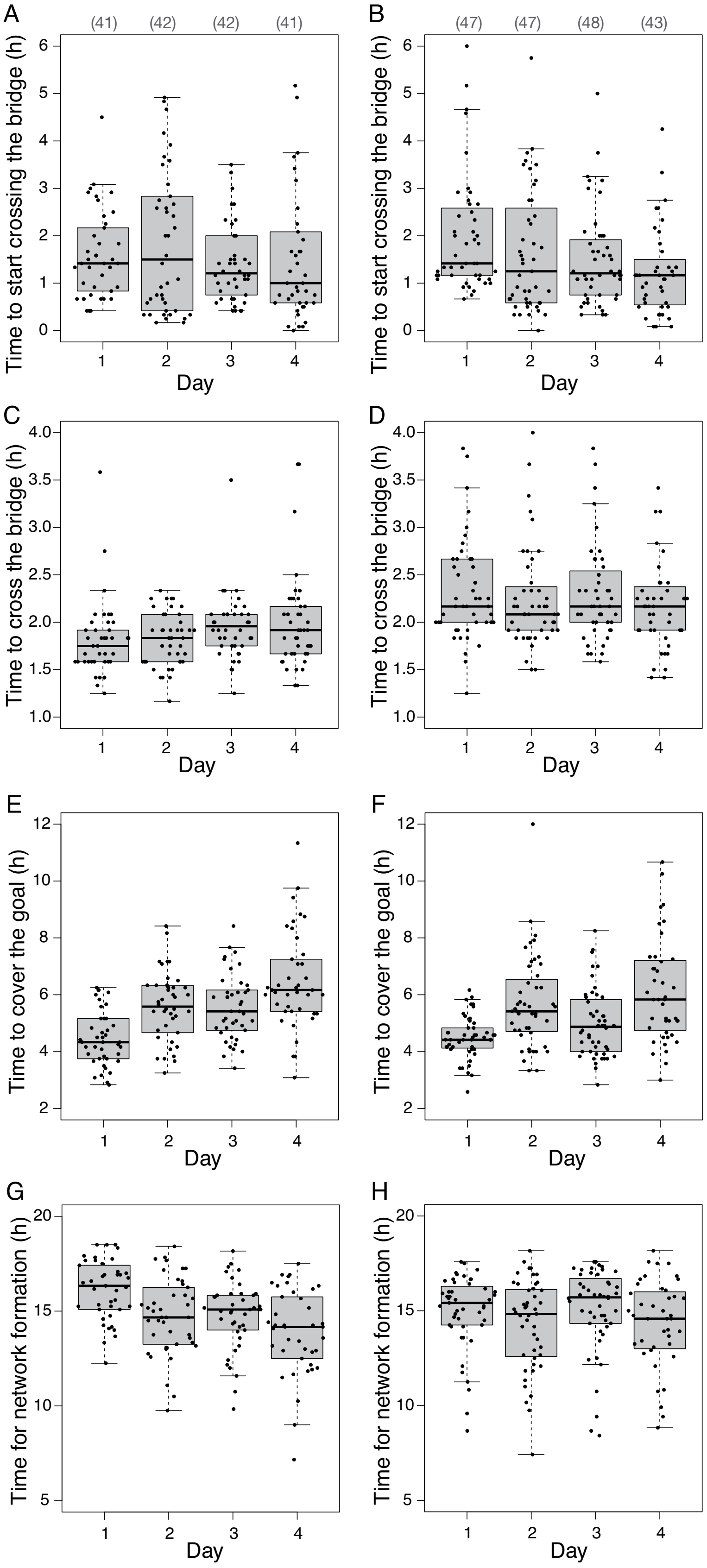

Figure S4. Periods of stages. (A, B) Time to begin to cross the bridge after start block setting. (C, D) Time for crossing the bridge. (E, F) Time for covering the goal. (G, H) Time for tube network formation. (A, C, E, G) Control experiment. (B, D, F, H) Quinine experiment. The numbers in parentheses indicate the number of data in each dataset.

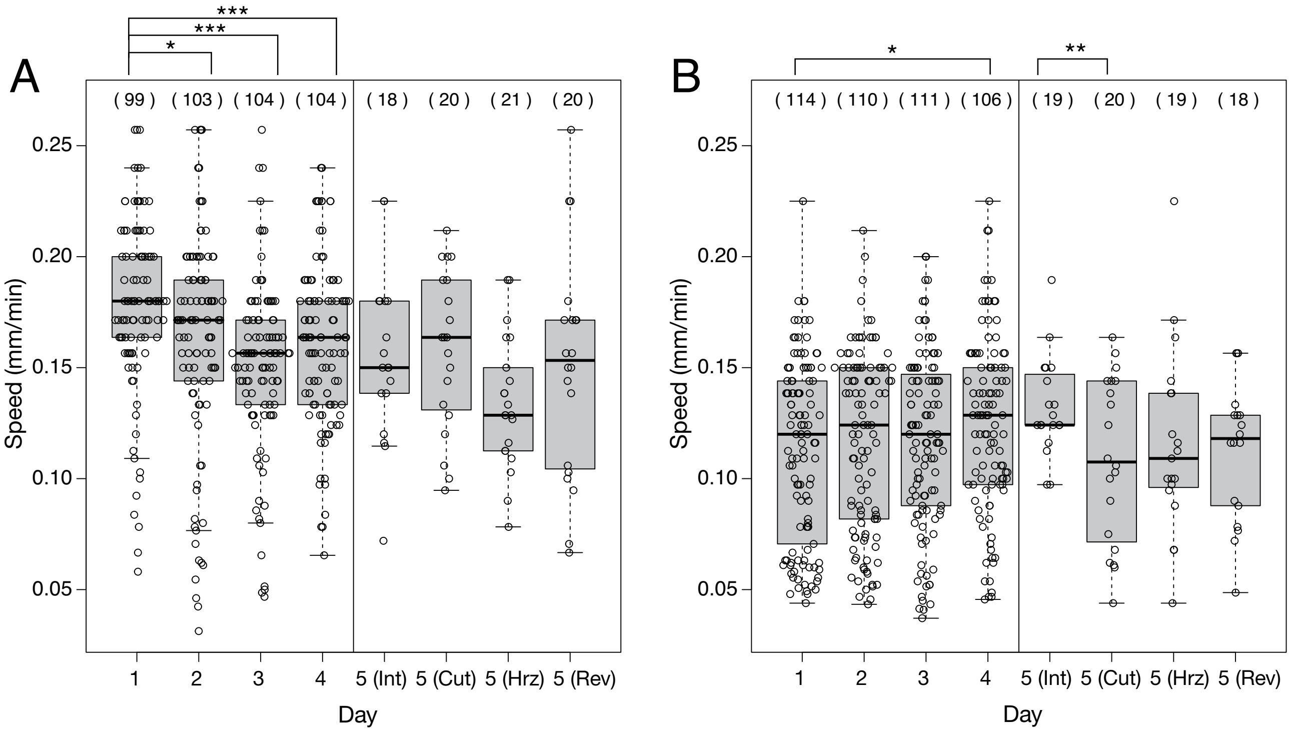

Figure S5. Speed of plasmodia crossing the bridge. (A) Control experiment. (B) Quinine experiment. The abbreviations in parentheses shown in day 5 represent Int: intact, Cut: cutoff, Hrz: horizontally placed, Rev: reversely placed. Open circles represent data of a single sample. Thick bars in box plots are the median of each dataset; Upper and lower edge of boxes are upper and lower quantiles, respectively; Upper and lower thin bars are the largest/smallest value less/greater than upper/lower quantile plus/minus 1.5 times interquartile range; see R manual for detail. The numbers in parentheses above the boxes indicate the number of data in each dataset. Asterisks represent the degree of p-value calculated as a result of appropriate statistical tests for each dataset as follows: * $p < 0.1$, ** $p < 0.05$, *** $p < 0.01$, and unlabeled $p > 0.1$. See Supplementary Table S1 for details. As reference data in the multiple comparison test, the Day 1 dataset was used for Days 1-4 of analysis, and the intact Day 5 dataset was used for Day 5 of analysis.

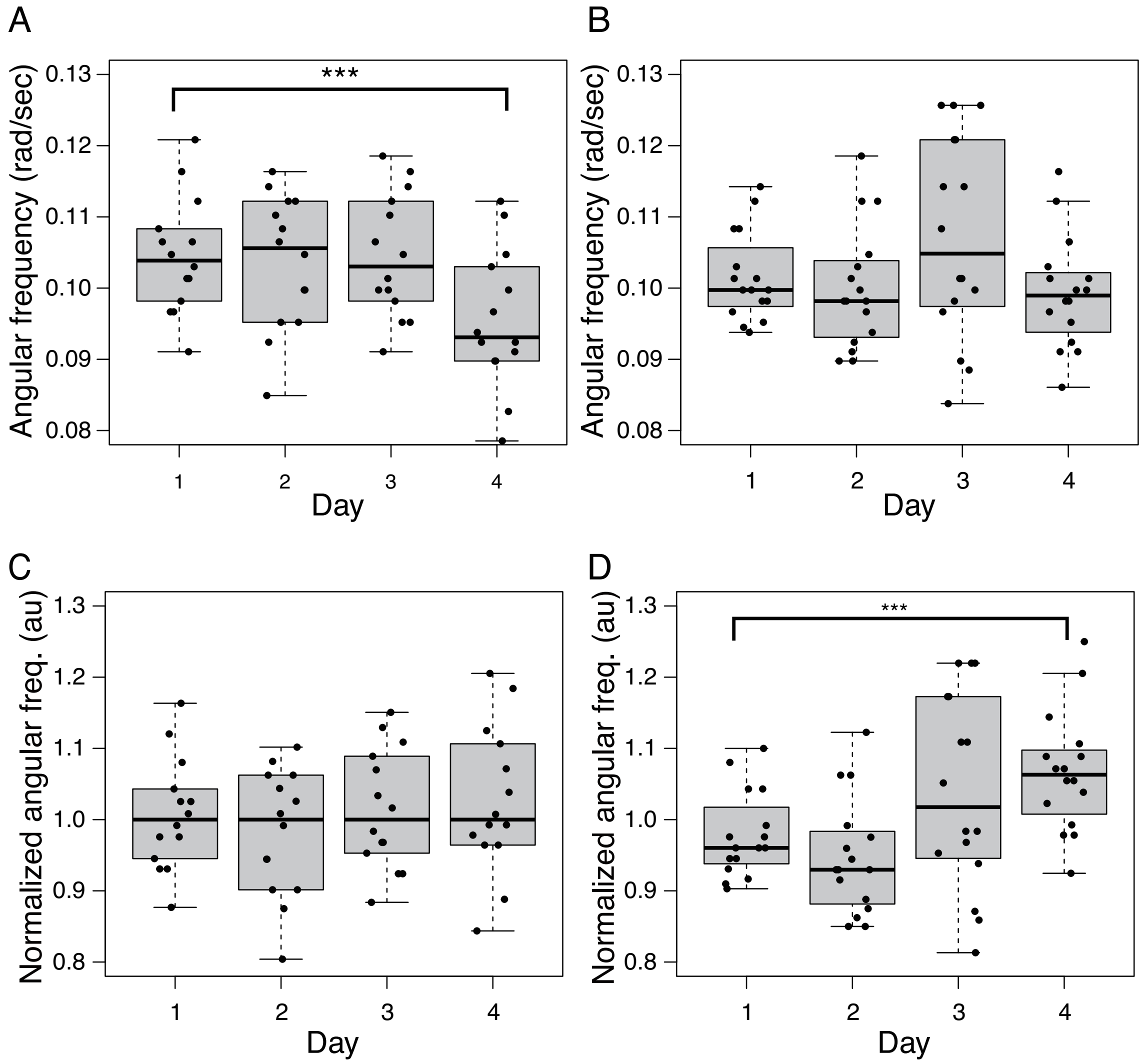

Figure S6. Angular frequency of thickness oscillation at start block. (A, C) Control experiment. N=14. (B, D) Quinine experiment. N=16. (A, B) Unprocessed data. (C, D) Data are normalized by the median of the control data on the same day. Asterisks represent the degree of p-value calculated as a result of appropriate statistical tests for each dataset as follows: *** $p < 0.01$, and unlabeled $p > 0.1$. As reference data in the multiple comparison test, the Day 1 dataset was used. See Supplementary Table S1 for details.

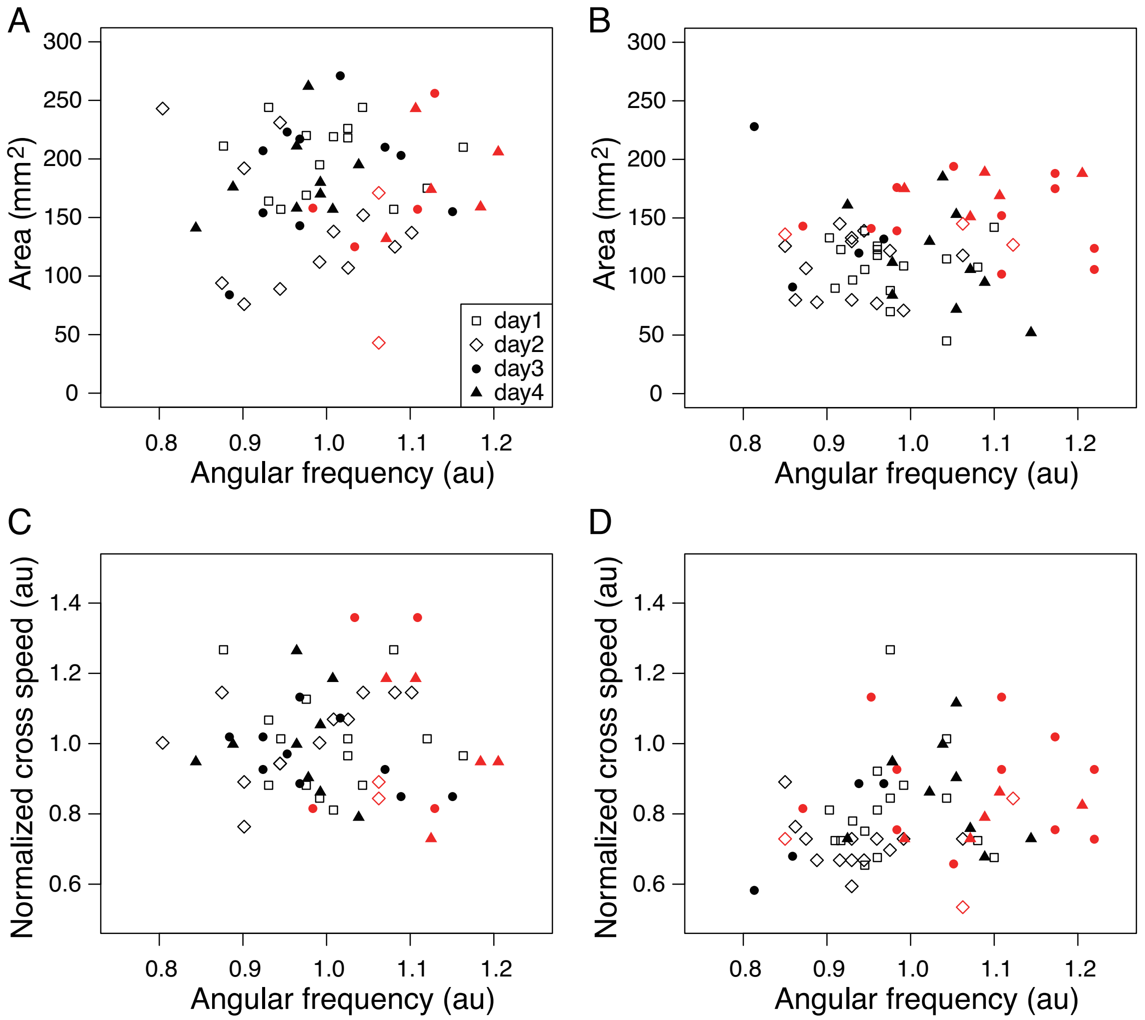

Figure S7. Relation between angular frequency and quantitative indices of habituation behavior. Angular frequencies are normalized by the median of the control data on the same day. (A, B) Relation between angular frequency and area. (C, D) Relation between angular frequency and normalized crossing speed. (A, C) Control experiment. (B, D) Quinine experiment. A legend for the symbols is shown in Figure A. The red and black colors correspond to the network type of mesh or tree at the start block, respectively, which are classified using the network morphologies formed at the previous day's goal. Correlation coefficients between two indices are 0.070 ($p = 0.61$) in Figure A, -0.016 ($p = 0.91$) in Figure B, 0.106 ($p = 0.41$) in Figure C, and 0.182 ($p = 0.15$) in Figure D.
